# The journey so far

**DOI:** 10.2349/biij.4.1.e1

**Published:** 2008-01-01

**Authors:** KH Ng, BJJ Abdullah, NA Kadri

**Affiliations:** 1 Department of Biomedical Imaging, Faculty of Medicine, University of Malaya, Kuala Lumpur, Malaysia; 2 Centre of Biomedical Engineering, University of Surrey, Guildford, Surrey, United Kingdom

With the start of this new year, ***biij*** has completed a two-and-a-half years of publishing. We wish to share some good news with you and also to keep you posted on our achievements since our last update in January 2006 [1].

## INDEXING

As part of our efforts in establishing ***biij*** as one of the important open-access journals dealing with topics relating to radiology, biomedical imaging and biomedical intervention, we have tirelessly pursued the indexing of ***biij*** in various relevant scientific indices and databases. For this reason, ***biij*** has been a member of CrossRef and has been made searchable by the Google Scholar search engine since it was first launched in July 2005. In that year, ***biij*** was only listed in the Directory of Open Access Journals (DOAJ). By the end of 2006, the journal was listed in and/or indexed by Chemical Abstracts Service, Inspec, Index Copernicus International, and eGranary Digital Library. In 2007, it was listed in the Open-J Gate web directory.

In December 2007, ***biij*** received the best news so far in its history of publication: being accepted for indexing by the Elsevier Bibliographic Databases as of 2008 onwards. In the confirmation letter from Elsevier, the inclusion of ***biij*** is “in recognition of the high quality and relevance (of its contents) to the scientific community”. The acceptance means that the contents of ***biij*** will now be available in Scopus, EMBASE, EMCare, Compendex, and several other specialised niche databases once the subscription processes have been completed.

The inclusion of ***biij*** in Scopus is particularly important, as it is one of the largest abstract and citation databases of research literature and web sources, and has developed a large worldwide customer base. This will certainly pave the way for the inclusion of ***biij*** in other important indexing services, particularly those provided by Thomson Scientific and National Library of Medicine.

## STATISTICS

The ***biij*** has published 10 issues thus far, with a total of 121 papers. These include four special focus issues: PET/CT and Molecular Imaging (Biomed Imaging Interv J 2006; Vol. 2, Issue 4, available at http://www.biij.org/2006/4/); Image-guided Surgery and Therapy (Biomed Imaging Interv J 2007; Vol. 3, Issue 1, available at http://www.biij.org/2007/1/); Radiation Dose Optimisation in Biomedical Imaging and Intervention (Biomed Imaging Interv J 2007; Vol. 3, Issue 2, available at http://www.biij.org/2007/2/); and Leadership and Management in Biomedical Imaging and Intervention (Biomed Imaging Interv J 2007; Vol. 3, Issue 3, available at http://www.biij.org/2007/3/).

The digital recording section continues to be very popular with the readers and offers great educational value. The number of recorded presentations rose from 32 when the journal was launched in 2005, to 121 in the year 2006, and to 146 in 2007. A number of meetings and conferences around the world have agreed to grant ***biij*** the exclusive rights to reproduce selected presentations to be shared with the journal’s readers and subscribers.

**Figure 1:**
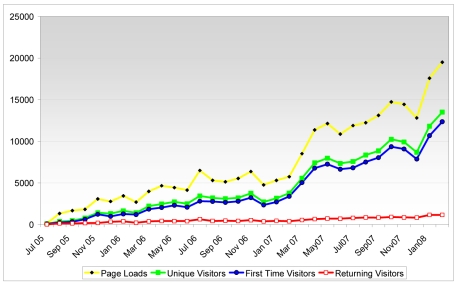
The monthly statistics of page loads, unique visitors and returning visitors for ***biij***, from July 2005 to December 2007.

Our readership has increased steadily over the years. When ***biij*** was first launched in July 2005, the website received a very modest 142 total hits, with only 8 returning visitors (Figure 1); but by November 2007, the journal recorded the highest traffic thus far in its short history of publication: a total of 14,438 hits with 846 returning visitors. Although there is no publicly available traffic data of other newly-launched peer-reviewed journal websites for a comparison to be made, the Editors truly believe that the increase is of considerable significance. The journal enjoyed an average of more than 100% increase in average monthly readership every year (Table 1). With the dawn of 2008, this trend is predicted to increase even more as proven by the latest traffic statistics: 12,351 total hits, with 840 returning visitors (as of 23 January 2008). Traffic statistics to ***biij*** were independently verified by both Statcounter (http://www.statcounter.com) and Google Analytics (http://www.google.com/analytics/) services.

**Table 1:** The summary of average monthly traffic statistics to ***biij*** website in 2005-2007.

**Year**	**Page loads**	**Unique visitors**	**First-time visitors**	**Returning visitors**
2005	1,796.7	730.5	573.7	156.8
2006	4,741.5 (+163.9%)	2689.6 (+268.2%)	2271.9 (+296.0)	417.7 (+166.3)
2007	11,093.8 (+134.0%)	7399.9 (+175.1%)	6700.1 (+194.9)	702.3 (+68.2)

The readership came from more than 100 countries around the world, thus confirming our founding decision to produce an open-access peer-reviewed journal that is freely accessible throughout the world. Of particular importance is an increasing number of readership and paper submissions from developing countries in Asia (including ex-Soviet bloc states), Africa, and South America. The record showed that the readers’ countries of origin were as wide-ranging as Italy to Indonesia, Portugal to Puerto Rico, Sweden to Sri Lanka, and United States to Uruguay.

As of December 2007, there were 219 submitted papers with a rejection rate of 29%. The average time between a submission date and the date it was first sent for review was 24 days, and the average processing time (from submission to acceptance) was 103 days. It took an average of 38 days for an article to be published following an acceptance.

## OPEN JOURNAL SYSTEMS (OJS)

**Figure 2:**
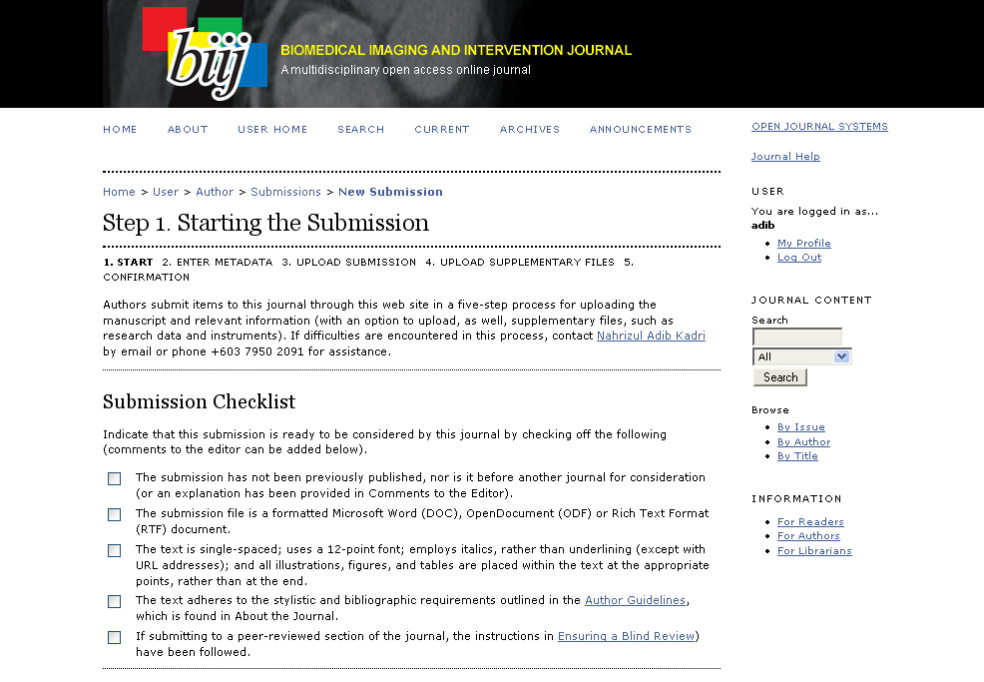
The online submission system of ***biij*** is powered by OJS.

At the end of 2006, ***biij*** gradually employed the use of an open source online submission system, the Open Journal Systems (OJS), developed and maintained by the Public Knowledge Project. The Project is a partnership between Faculty of Education at the University of British Columbia, the Simon Fraser University Library, the School of Education at Stanford University, and the Canadian Centre for Studies in Publishing at Simon Fraser University [2]. The OJS (Figure 2) allows for an easier and more streamlined peer-review and publishing process, both for the reviewers and the editorial staff. Its features are comparable to the other proprietary paper submission systems available in the market today, particularly in allowing the authors to track the progress of their submitted papers.

## OUR PLAN

We have initiated the application process for the inclusion of ***biij*** in the United States National Library of Medicine’s PubMed® (http://www.pubmed.gov/), particularly in its largest component, the MEDLINE®. Currently about 5,000 biomedical journals from around the world are indexed in MEDLINE [3], and it is generally accepted as the primary bibliographic indexing service for biomedical journal citations and abstracts worldwide.

Along with this effort, the contents of ***biij*** are also currently being evaluated for inclusion in the PubMed Central (http://www.pubmedcentral.nih.gov/), the United States National Institutes of Health (NIH) digital archive of biomedical and life sciences journal literature. This move is primarily due to PubMed Central being the only acceptable public repository of journal contents by the U.S. National Library of Medicine. This effort should be completed before the end of the year, as it involves purely technical processes of preparing fully-tagged XML data of all published ***biij*** contents.

Apart from successful inclusion in PubMed Central, a more pressing need towards achieving the PubMed® listing is in maintaining the quality of published articles in ***biij*** at a continuously high level and in a timely manner. This has been one of the more challenging issues faced by ***biij*** over its two years of publication. Nevertheless, with continuous support from a dedicated team of editorial staff, we will ensure that these challenges are taken up and resolved as efficiently as possible.

We take this opportunity to thank our authors, reviewers, sponsors, and readers who have been supporting us since day one. Your continuous support is the reason ***biij*** is where it is at today, and we are definitely counting on you to help us scale greater heights tomorrow.

Let us continue with our journey.
